# From ear to hand: the role of the auditory-motor loop in pointing to an auditory source

**DOI:** 10.3389/fncom.2013.00026

**Published:** 2013-04-22

**Authors:** Eric O. Boyer, Bénédicte M. Babayan, Frédéric Bevilacqua, Markus Noisternig, Olivier Warusfel, Agnes Roby-Brami, Sylvain Hanneton, Isabelle Viaud-Delmon

**Affiliations:** ^1^STMS IRCAM-CNRS-UPMC, IRCAMParis, France; ^2^Laboratoire de Neurophysique et Physiologie, CNRS UMR 8119, UFR Biomédicale des Saints Pères, Université Paris DescartesParis, France; ^3^Institut des Systèmes Intelligents et de Robotique, CNRS UMR 7222, UPMCParis, France

**Keywords:** spatial audition, human, pointing movement kinematics, orienting movements, reaching, auditory-motor mapping, movement sonification

## Abstract

Studies of the nature of the neural mechanisms involved in goal-directed movements tend to concentrate on the role of vision. We present here an attempt to address the mechanisms whereby an auditory input is transformed into a motor command. The spatial and temporal organization of hand movements were studied in normal human subjects as they pointed toward unseen auditory targets located in a horizontal plane in front of them. Positions and movements of the hand were measured by a six infrared camera tracking system. In one condition, we assessed the role of auditory information about target position in correcting the trajectory of the hand. To accomplish this, the duration of the target presentation was varied. In another condition, subjects received continuous auditory feedback of their hand movement while pointing to the auditory targets. Online auditory control of the direction of pointing movements was assessed by evaluating how subjects reacted to shifts in heard hand position. Localization errors were exacerbated by short duration of target presentation but not modified by auditory feedback of hand position. Long duration of target presentation gave rise to a higher level of accuracy and was accompanied by early automatic head orienting movements consistently related to target direction. These results highlight the efficiency of auditory feedback processing in online motor control and suggest that the auditory system takes advantages of dynamic changes of the acoustic cues due to changes in head orientation in order to process online motor control. How to design an informative acoustic feedback needs to be carefully studied to demonstrate that auditory feedback of the hand could assist the monitoring of movements directed at objects in auditory space.

## Introduction

Interactions between the auditory and motor systems are mainly studied in the context of musical rhythm or vocal sounds perception and production (e.g., Hickok et al., 2003; Chen et al., 2009). However, hand pointing to sounds is often used to study auditory localization. It is a complex task that relies on a precise representation of auditory space that can be used for the control of directional motor output. Just like pointing to visual targets, it involves different modular neural processes since spatial information about the target position and hand position have to be combined across different senses and reference frames.

In order to address the mechanisms whereby an auditory input is transformed into a motor command, we studied online auditory control of the direction of pointing movements toward auditory sources. We first investigated whether pointing movements were more accurate when the target was present throughout the entire pointing movement than when the target disappeared shortly after the hand movement had begun.

We then added an auditory feedback of the pointing hand's position during the entire hand movement to evaluate whether human subjects could use such a feedback. This additional auditory feedback named auditory avatar (by analogy with avatars used to represent visually a part of the body of a participant in a virtual environment) was used in order to evaluate whether it would constitute stable and relevant information to guide the motor action of the user, as already suggested by recent results indicating that auditory information is used to control motor adaptation (Oscari et al., 2012). With such an auditory feedback, the auditory modality conveys supplementary sensory information that is correlated with proprioception and set in modular processes in the same spatio-temporal reference frame as the target, hence facilitating precision in the pointing task. A well-designed auditory avatar, which corresponds to a sonification transforming relevant parameters of human movement patterns into appropriate sound, could be used to enhance perception accuracy and would be useful for sensory substitution and motor training technologies.

The first auditory avatar condition was contrasted to a shifted condition where the heard hand position did not correspond to the actual hand position thus resulting in a discrepancy between auditory and proprioceptive information. Similar methodology can be found in Forma et al. (2011), where participants were asked to point to virtual targets in a spatialized audio environment using the openAL library (interaural time and level differences based audio environment). Studying online adaptation to this sensory conflict was expected to provide further information about the contribution of auditory inputs generated by arm movements to motor control.

## Materials and methods

### Subjects

Twenty-four self-reported right-handed volunteers (12 females and 12 males; 25.6 ± 6.6 years old) participated in the experiment. All were healthy and had normal hearing. The study was carried out in accordance with the Declaration of Helsinki. All subjects gave written informed consent and were paid for their time.

### Experimental setup

The experiment used real-time controlled virtual audio rendering for both representing sound sources at the target positions in space and attaching sounds to the subject's right hand during the pointing movement. Audio was played back over headphones and subjects were seated in front of a table from which the auditory targets virtually originated. To prevent any visual input interference during the experiment all subjects were blindfolded.

**Figure 1:**
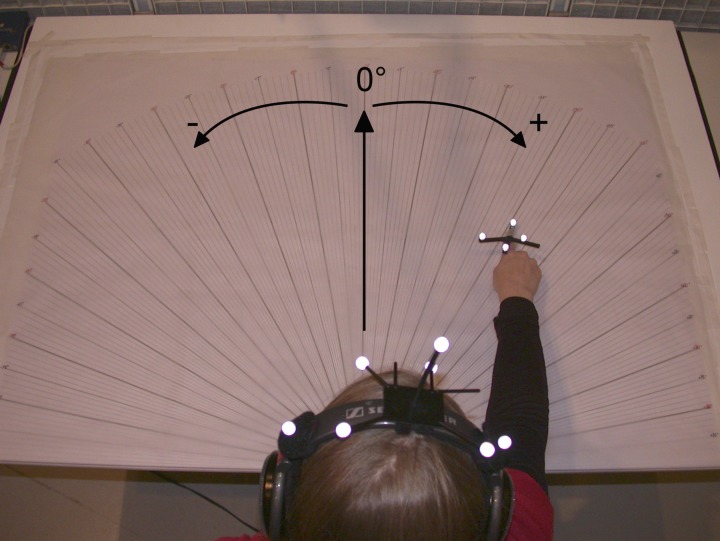
**View of the experimental set up, protractor on the table (0° axis straight ahead) and optical markers of the Optitrack 3-D motion capture system on the head (attached to the headphones) and right hand of the subject.** Note the positive/negative angles reference.

The stimuli for target sources and the auditory avatar were (mutually uncorrelated) white Gaussian noise signals. The virtual audio targets as well as the auditory feedback of the hand position were provided with the Head-Related Transfer Functions (HRTFs) binaural technique (Wightman and Kistler, 1989a,b). Spat~, IRCAM's software for real-time sound source spatialization, was used to create the binaural signals. Binaural rendering uses HRTFs to reproduce the sound pressure at the ear entrance that corresponds to a sound source at a given position in 3-dimensional space. Processing a monophonic audio signal with a set of HRTF filters and playing these signals back over headphones creates the illusion of a virtual sound source at the corresponding position in space. The spatialization of the sounds (stimuli and hand position) was calculated in real-time through the tracking of the head's and right hand's positions and orientations using a six-camera Optitrack (by Natural Point) 3-D infrared motion capture system. To this end, two rigid sets of markers were placed on the headphones and the right-hand's forefinger. They were, respectively, composed of seven and four reflective markers tracked by the cameras. The coordinates of the hand and head's locations in space were measured and recorded with the tracking system at a sampling frequency of 100 Hz. The minimal latency of the overall system is then 10 ms, with an audio latency of 0.6 ms, which is fast enough to ensure perceptive coherence when localizing virtual sound sources (Brungart et al., 2004). The orientation of the 7-marker rigid body fixed to the headphones allowed for computing the heading direction (0° is forward, positive is to the right, see Figure 1). The endpoint used to measure the kinematics of the hand corresponded to the tip of the index finger.

### Experimental procedure

The experiment lasted 1 h and was composed of pre-trials and 4 sessions. The pre-trials aimed at selecting the best-fitting HRTF from a set of several HRTFs. This best-fitting HRTF was then used to convolve the stimuli of the main experiment. Subject tested HRTFs previously selected in HRTFs fitting past experiments [see Sarlat et al. (2006) for a description of the method] plus their individual HRTFs when available, while hearing the spatialized targets. Up to four functions were tested. Approximately 10 practice trials per tested HRTF were performed in a pseudo-random order using the five targets of the experiment. Subjects were asked if they heard a spatialized sound and if so were asked to point toward its direction. The HRTFs were selected if in at least 8 trials the subjects pointed toward the correct direction (±10° approximately). The five subjects who did the pre-trials with their own HRTFs used them. The other subjects did not have individual HRTFs and used the non-individual HRTFs they selected during the pre-test.

Each session tested a different condition. In the short sound condition (named A) the auditory target was played for 250 ms before subjects pointed toward it. In the long sound condition (B) the auditory target was played for 2000 ms and subjects pointed toward it whilst hearing the auditory stimulus. Two other sessions included the auditory avatar that provided auditory feedback of the position of the hand in space. The fingertip position was dynamically tracked in real-time with the motion capture system and controlled the sound spatialization. Thus the white Gaussian noise stimulus was perceived as coming from the hand position. In these sessions the target was displayed during 250 ms and the avatar was heard constantly. In the “avatar condition” the actual hand position was heard (C), and in the “conflicting avatar condition” (D) the audio rendered hand position was shifted 18.5° left from the real hand position. Before each session, the subjects did a few trials to get used to the task demands and to the auditory feedback. The subjects were divided into 2 groups: group 1 performed the sessions in the regular order (A-B-C-D) and group 2 in the reverse order (D-C-B-A).

At the beginning of a trial, subjects were told to put their right hand on the table in front of them near their abdomen, with the palm at a position indicated by a tactile marker, and to hold their head up right facing ahead during the experiment. The auditory sources originated from a virtual distance of 60 cm in the horizontal plane of the table centered by the tactile marker. The targets originated from five directions with azimuth angles of −35°, −20°, 0° (ahead of the subject), 20°, and 35° (right is positive). Each session contained 32 trials presented in the same pseudo-random order for each subject. Moreover, the table on which the subjects pointed was covered with a semi-circular protractor of which origin was located at the starting hand position. It enabled a measure in degrees of the pointing as subjects were asked to keep their hand still for a few seconds after pointing. After each trial the subjects put their hand back to the tactile marker. The experimental setup from the subject's viewpoint is shown in Figure 1.

## Data analysis

### Level of performance

The pointing direction was directly measured on the protractor. The level of performance is evaluated by the signed angular error, which is the difference between the target direction and the final direction pointed by the subjects. If the subject pointed to the left of the target, the error was negative, and conversely it was positive if the subject pointed to the right of the target.

### Movement analysis

**Figure 2:**
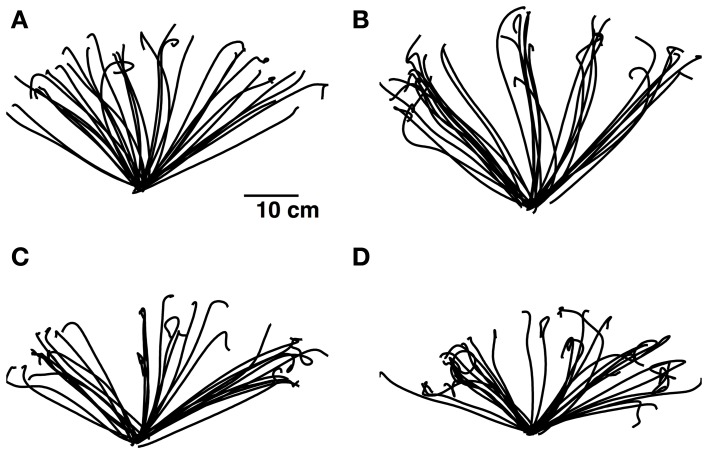
**Typical trajectories of the tracked hand for a single subject for each of the four conditions tested: short sound condition (A), long sound condition (B), avatar condition (C), and conflicting avatar condition (D).** Better pointing precision and reduced overshooting is noticeable in condition **(B)**.

The raw data of hand and head positions was recorded and processed off-line for the analysis of the kinematics of hand and head movements. A semi-automatic method was designed to detect and segment each pointing gesture and eliminate the way back to the start tactile marker. A primary segmentation was performed by applying thresholds on the hand displacement along the horizontal plane (*x, y*). The typical trajectories projected on the horizontal plane are shown in Figure 2 for each condition.

**Figure 3:**
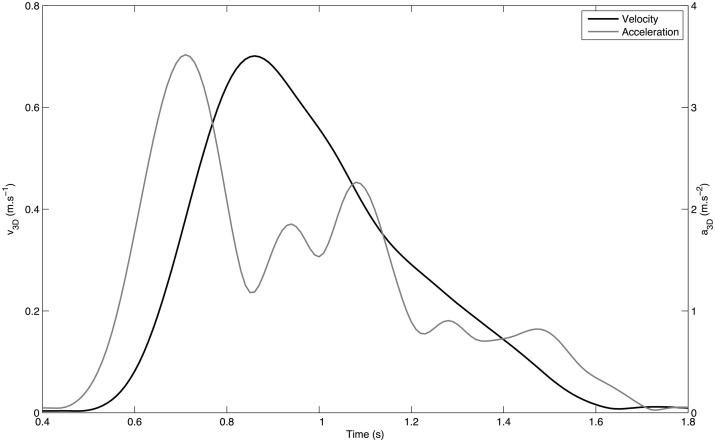
**Typical tangent velocity v3D (bold line) and a3D (gray line) profiles of a pointing movement**.

The second segmentation process was based on systematic movement kinetics analysis. To compute velocity, acceleration and jerk, position data was filtered with a Gaussian low-pass filter, with a cut-off frequency of 5 Hz. As the movement is captured along the 3-dimensions of space the computed values are 3-dimensional energy-related vectors: *v*_3*D*_, *a*_3*D*_, and *j*_3*D*_ are, respectively, the norms of the tangential velocity, acceleration and jerk vectors. The beginning and the end of movement were defined as the crossing of a threshold on *v*_3*D*_ corresponding to 3% of the peak velocity calculated on the trajectory. The “beginning” of the gesture is thus related to the energy of the movement. The typical velocity and acceleration profiles obtained for one pointing gesture are plotted on Figure 3.

Additionally, kinematic analysis included the following measures for hand and head movement: movement duration, peak velocity value, average velocity, acceleration peaks analysis (occurrence and position), and trajectory length in space. We counted the total number of acceleration peaks occurring before and after the maximum velocity peak of the movement (peak velocity point PVP).

In order to investigate the possible role of the head in sound localization before and during pointing to the estimated location of the source, we also measured the heading angle around the vertical axis and computed its maximum values and range of motion (ROM).

## Results

### Statistical analysis

The results of six participants were removed from the analysis based on three criteria: subjects who did not follow the instruction to point directly toward the target (the trajectory duration is more than twice the average and longer than the longer stimulus duration in the long condition), three subjects; trajectories showing no dependence on the target direction (with only two ±90° endpoints), two subjects; short trajectories (less than 10 cm) that lead to unstable angular calculations, one subject.

The dependent variables considered in our statistical analysis (ANOVA) are the averaged measures (duration, maximum velocity, average velocity etc.) over each target direction and each condition. In the statistical analysis, we considered two grouping factors. The first is the two-level HRTF factor that indicates if the subject used his own HRTF or not. The second factor is the two-level group factor that indicates the order of the presentation of the experimental conditions. We also considered two repeated-measure factors. The first one, the five-level target direction factor, corresponds to the direction of the target. The second one is the four-level condition factor indicating the experimental condition of each trial (A-B-C-D).

Statistical data analysis showed no main effect of the group factor. There was thus no effect of the order of the conditions either on the pointing performance or on the dynamical control of the gestures. There was a main effect of the individualized HRTF only on the proportion of acceleration peaks of the head after the PVP [*F*_(1, 16)_ = 5.8, *p* < 0.05]. However the average peak number was not significantly different between the two-levels of the HRTF factor (*post-hoc* Bonferroni test). It is important to note that the individualized HRTF factor had no effect on the measures related to hand movement. The group factor and the individualized HRTF factor will not be used further in the analysis and data will be averaged per factor.

### Level of performance

**Figure 4:**
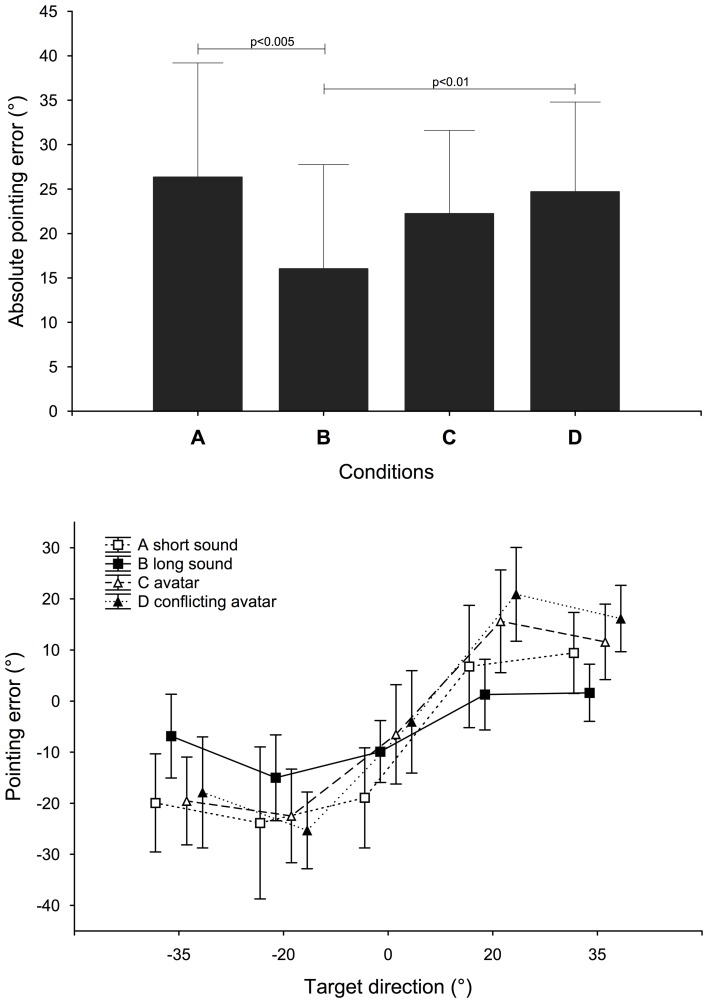
**Absolute pointing error in degrees (absolute difference between pointed direction and target direction) for each condition and signed pointing error in degrees (difference between pointed direction and target direction) for each target direction and each condition.** Target direction goes from left (negative) to right (positive). A positive error indicates a pointing to the right of a target. Bars indicate 95% confidence interval.

There was a main effect of the condition factor but also of the target factor on the absolute value of the angular error [*F*_(3, 51)_ = 6.23, *p* < 0.005 and *F*_(4, 68)_ = 5.80, *p* < 0.001, respectively]. Subjects were significantly more accurate in the long sound condition B (see Figure 4 top which shows the absolute pointing error for the different conditions and the results of the *post-hoc* Bonferroni test; error bars indicate 95% confidence interval). Furthermore, there was a significant interaction between the two factors [*F*_(12, 204)_ = 1.91, *p* < 0.05].

We also analysed the signed angular error as the sign indicates if the subjects pointed more to the left or more to the right of the target direction. There was a main effect of the condition [*F*_(3, 51)_ = 2.84, *p* < 0.05] and the target direction factor [*F*_(4, 68)_ = 20.34, *p* < 0.0001], and there was a significant interaction between condition and target direction factors [*F*_(12, 204)_ = 6.13, *p* < 0.0001]. Targets' azimuths were over-estimated by the subjects (see Figure 4 bottom which shows the signed pointing error for target directions and among conditions tested). Left targets were pointed with negative errors, and right targets with positive errors. This overshooting was reduced in the B condition: −66%, −40%, −48%, −94%, and −90% for targets from left to right compared to the maximum errors in the other conditions. However, it is important to note that the subjects still presented a 9.8° average bias on the left when the target was presented straight ahead in the B condition.

### Global kinematics

The parameters associated with movement velocity were significantly influenced only by the target direction [*F*_(4, 68)_ = 8.66, *p* = 0.00001 for the duration; *F*_(4, 68)_ = 81.59, *p* < 0.00001 for the peak velocity and *F*_(4, 68)_ = 72.31, *p* < 0.00001 for the average velocity]. Peak and average velocities were significantly higher for target sounds coming from the right (i.e. for +20° and +35°): +37% for peak velocity and +31% for average velocity, *post-hoc* Bonferroni test *p* < 0.0001. The same test revealed no exploitable difference between the five target directions regarding movement duration.

**Figure 5:**
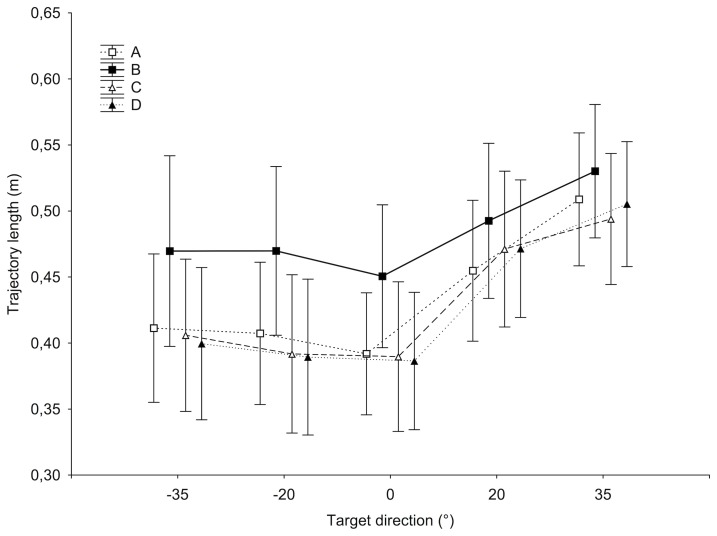
**Trajectory length in space (in meters) for each target direction and each condition.** Bars indicate 95% confidence interval.

The condition factor, the target direction factor and their interaction had a significant effect on the trajectory length [*F*_(3, 51)_ = 5.47, *p* < 0.005; *F*_(4, 68)_ = 47.03, *p* < 0.0001 and *F*_(12, 204)_ = 2.58, *p* < 0.005, respectively]. The analysis showed a significantly longer distance covered for targets on the right (0.473 m at +20°, 0.510 m at +35° against 0.414 for the three other targets averaged, *p* < 0.005), but also in the B condition (0.482 m against 0.432 m on average, *post-hoc* Bonferroni test *p* < 0.05; see Figure 5).

### Movement dynamics and segmentation

The counting of acceleration peaks revealed a significant effect of condition factors [*F*_(3, 51)_ = 3.04, *p* < 0.05] and target direction [*F*_(4, 68)_ = 30.93, *p* < 0.00001] on the total number of peaks and on the proportion of peaks before reaching the PVP [*F*_(3, 51)_ = 3.34, *p* < 0.05 and *F*_(4, 68)_ = 36.97, *p* < 0.00001].

In the B condition, subjects' movements presented larger total number of acceleration peaks, however, not significantly different from the other conditions (4.85 against 4.20 on average).

The number of peaks decreased as the target direction shifted to the right of the subjects (significantly for the two targets on the right, *post-hoc* Bonferroni test *p* < 0.0005: 3.89 and 3.70 against 4.73 on average). Only the target direction factor had an effect on the proportion of peaks after PVP [*F*_(4, 68)_ = 10.68, *p* < 0.00001] significantly different for +20° and +35° targets (−18% at +20° and −22% at +35° on average), while there was a marginally significant effect of the condition factor [*F*_(3, 51)_ = 2.47, *p* = 0.07].

It is noticeable that subjects produced movements with more acceleration peaks on the second “half” of the trajectory, during the deceleration phase: 1.52 before the PVP, 2.84 after on average. If taken as a factor, the proportion of peaks before or after PVP together with the condition factor shows a significantly higher increase of peaks after PVP for condition B than conditions A and C (*post-hoc* Bonferroni test *p* < 0.01).

### Head movement analysis

The same analysis was conducted on the head movement data. The target direction factor had a significant effect on the total number of acceleration peaks in the head movement [*F*_(4, 68)_ = 5.75, *p* < 0.005] with the same tendency toward right directions as for the hand (7.14 peaks for −35°, 6.53 for +35°). No significant effect was found on the proportion of acceleration peaks before PVP. After this point, both target direction and condition factors have significant effects [*F*_(4, 68)_ = 4.97, *p* < 0.005 and *F*_(3, 51)_ = 6.93, *p* < 0.001, respectively], again with the same behavior as for the hand. The B condition exhibited a significantly larger numbers of peaks after PVP (+50% for B on average, *post-hoc* Bonferroni test *p* < 0.05) than in the other conditions and the center and right targets exhibited fewer acceleration peaks (−17% on average).

**Figure 6:**
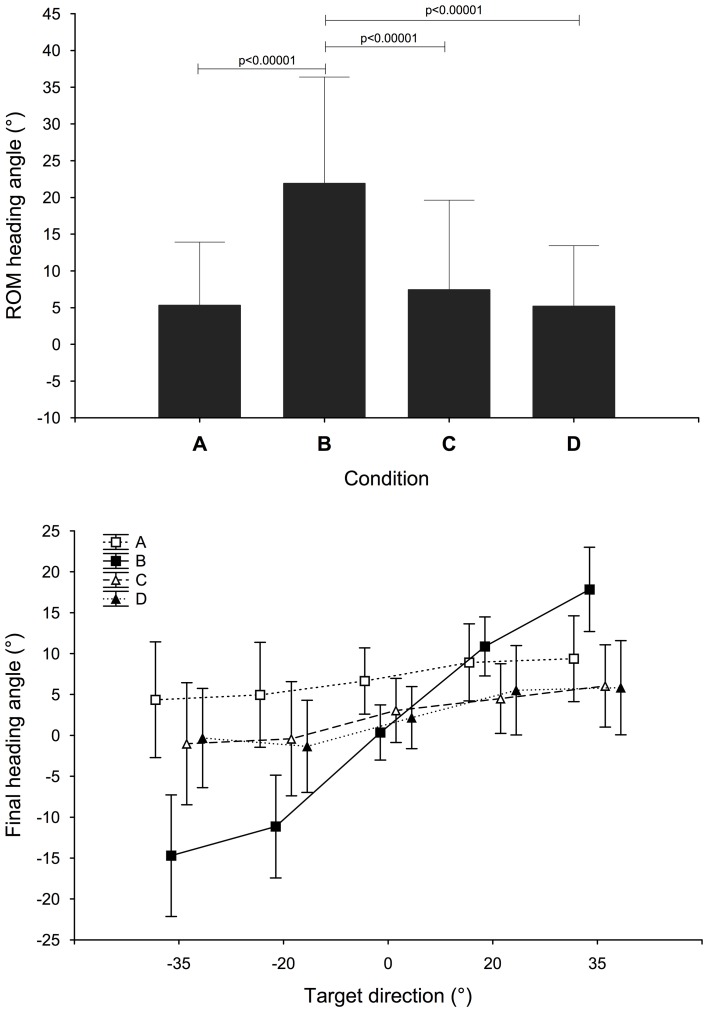
**Range of motion of the heading angle (in degrees) for each condition.** Effect significance: [*F* = 20.2, *p* < 0.0001]; *post-hoc* Bonferroni test; Final heading angle (in degrees) for each target direction and each condition. Interaction effect significance: *F* = 14.9, *p* < 0.00001. Bars indicate 95% confidence interval.

Both condition and target direction factors had a significant effect on the ROM of the heading angle [*F*_(3, 51)_ = 20.2, *p* < 0.0001; *F*_(4, 68)_ = 3.93, *p* < 0.01 respectively] and there is a significant interaction between the two factors [*F*_(12, 204)_ = 2.40, *p* < 0.01]. The ROM of the heading angle was significantly higher in the B condition than in the other conditions (21.9° against 5.31°, 7.42° and 5.17° for A, C, and D conditions, *post-hoc* Bonferroni test), as shown in top Figure 6. No significant difference was found among the target directions but the ROM increased with the target eccentricity (+45% on the left, +23% on the right on average compared to 0° target).

In order to investigate the potential link between target direction and head rotation for localization when pointing we analysed the distribution of the heading angles at the end of the movement. As for the ROM, the condition factor, target factor and their interaction had an effect on the angle [*F*_(3, 51)_ = 5.07, *p* < 0.005; *F*_(4, 68)_ = 9.17, *p* = 0.00001 and *F*_(12, 204)_ = 14.9, *p* < 0.00001 respectively]. Significant differences were found for the two right targets compared to left targets (*p* < 0.01); the subjects turned their head toward the correct hemisphere corresponding to the target direction. When coupling the effect of the condition and the target direction, we found that this behavior was prevailing under condition B (see Figure 6 bottom). The two graphs on Figure 6 show that subjects moved their head more under condition B and in the direction of the target. The bias for 0° target is also reduced under this condition: 0.30° compared to 6.66° for A, 3.06° for C, and 2.18 for D.

**Figure 7:**
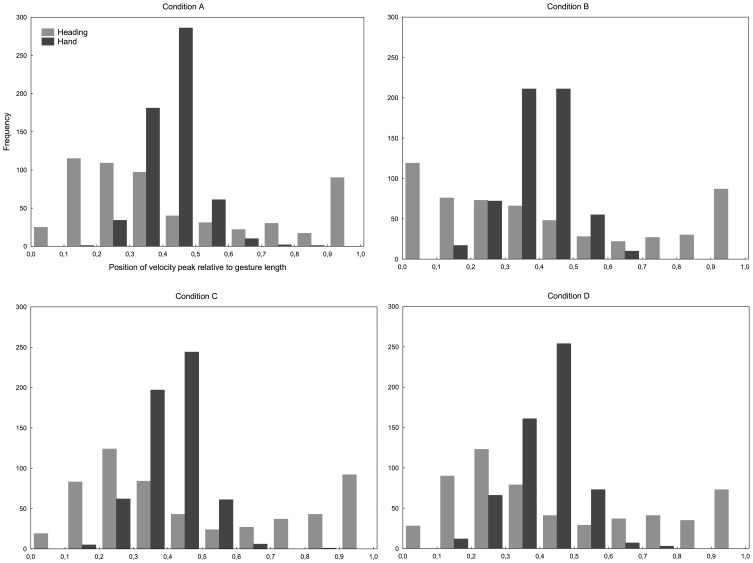
**Distribution of the position of the peak velocity point of heading angle and hand relatively to gestures length for every trial over every subject in each condition: short sound condition (A), long sound condition (B), avatar condition (C), and conflicting avatar condition (D).** It indicates that head maximum velocity is reached sooner than for the hand.

The analysis of the relative position of the PVP along the movement of the hand and the heading angle shows that subjects tended to initiate the movement of their head before the pointing movement. The distribution of these relative positions is shown in Figure 7 for every trial over every subject in each condition. On average, 43% of the gestures exhibited heading peak velocity between the beginning and the first third of the movement completion against 12% only for the hand. The tendency is observed in all the conditions and in spite of the large differences in ROM of heading and final angle between conditions.

## Discussion and conclusion

In this study, we attempted to address the mechanisms whereby an auditory input is transformed into a motor command. First, we aimed at assessing the role of auditory information about target position in correcting the trajectory of the hand by varying the duration of the target presentation. Second, we attempted to evaluate whether human subjects could use an auditory feedback about their hand position and how they would react to shifts in this avatar of their heard hand position.

Only the long sound target condition exhibited a higher level of performance of the subjects. This strong effect is comparable to the one obtained during pointing movements toward visual targets present throughout the entire pointing movement (Prablanc et al., 1986). In the present study, the target is presented during the whole movement only in the long sound duration condition (B). In the short sound duration condition, the location of the target needs to be memorized and it is possible that a shorter sound would lead to a less precise or reliable representation of the target. Errors in pointing to remembered targets presented visually have been shown to depend on delay between target offset and pointing (McIntyre et al., 1998). Therefore, the neural processes involved in coding the target in a motor-related or body-related reference frame from its auditory spatial trace seem to require a sufficiently long auditory stimulation. On the other hand, one can assume that comparison of auditory information about target position with proprioceptive information is required to update or refresh an internal representation of the goal to drive optimally the pointing hand.

In addition to better performance and precision (reduced bias for 0° target), subjects presented longer trajectories in the longer sound condition and slightly more acceleration peaks. The proportion of acceleration peaks in the deceleration part of the movement also increased in this condition. These results show that the improvement of precision in this condition may not only be due to better memorization of the target but also to the possibility to make online corrections of the hand trajectory. The use of auditory information about target direction as a feedback for guiding the reaching movement is likely since the kinematics showed indices of iterative corrections in condition B (in particular, increased length of the trajectory and increased number of peaks after PVP). These online corrections can be produced only if a neural process is able to use the auditory estimation of the target position and to make it available continuously to the sensorimotor process that drives the hand. Therefore, a sound still heard at the end of the pointing movement as in condition B would allow a more efficient updating of the goal representation in relation to the hand's position and thus a more accurate movement.

### Contribution of the auditory avatar

As demonstrated in Oscari et al. (2012), hand trajectory can be controlled and optimized with an auditory feedback. Here, the directional accuracy of pointing movement was not greater with auditory feedback of the hand position than without this information available (comparison of conditions A and C). Furthermore, in condition D auditory feedback of hand position was shifted by 18.5° perpendicularly to the main movement direction. Following the shift, the hand trajectory was expected to deviate from those produced in the condition without the shift. The analysis showed no significant effect of the resulting discrepancy between auditory and proprioceptive information about hand position on the pointing accuracy. It is possible that the levels of performance in all conditions but the long target condition were impeded by an inaccurate representation of the target relative to the body and that this important inaccuracy masks a small effect of the hand auditory feedback. Indeed, in the short sound condition with no avatar (A), the mean absolute pointing error was of 26°, higher than the shift used with the avatar in condition D.

In the avatar conditions, the proprioceptive modality also might have overtaken or dominated the overflowed auditory modality, hence the importance of the design of such feedback, as showed in Rosati et al. (2012). In their study, the authors compare the contribution of different sound feedbacks on the performance in a manual tracking task and their interaction with visual feedback. They have observed that sound feedback can be counterproductive depending on the task and mapping between gesture and sound. In our experiment the same sound was used for the targets and the hand feedback. This might have confused the subjects when localizing the target and addresses the question whether spatial auditory information about limb position is enough to provide an efficient feedback to a motor action. Different parameters of the motor action might indeed need to be sonified (for instance kinematics rather than position in space). It is therefore important to study the appropriate parameters for auditory-motor mapping before being able to provide useful information for rehabilitation and sensory substitution devices.

### Head movements

The analysis of final head orientation showed that in B condition heading automatically accompanied the auditory-manual pointing task despite the explicit instruction to avoid head movements. Thus, head rotations were only present when sufficient localization cues were available and the heading direction was consistently related to target direction and eccentricity. The first hypothesis than can be proposed is that this result indicates that in all the other conditions tested, the auditory target was too short to provide enough information to elicit head movements. However, since the heading direction and the direction of the pointing are clearly related in condition B (see Figure 6), one can propose also that the long sound allows an orienting movement of the head toward the auditory target and that the final angle of this orienting movement could guide the pointing movement of the hand. The fact that the head tends to achieve its maximum heading velocity before the hand PVP in all the conditions (see Figure 7) shows that early movement of the head alone did not lead to improved performance in condition B, but did along with a larger ROM and heading toward the target.

In general, heading movements belong to automatic orienting reactions that have been mainly studied in the framework of gaze orienting behavior (Guitton, 1992). Here in blindfolded subjects, we can assume that heading also aims at optimizing the binaural perception of the acoustic stimulation direction. The auditory system certainly relies on head motor information to build representations of the location of auditory targets. However and unfortunately, sound localization is mainly studied with the head fixed. Nevertheless several studies have used head orientation to quantify the ability of participants to indicate the perceived direction of a natural acoustic stimulation (Perrott et al., 1987; Makous and Middlebrooks, 1990; Pinek and Brouchon, 1992). These studies demonstrated that the direction indicated by the head was underestimated (~10°). We obtained similar results despite different experimental conditions (voluntary head pointing vs. automatic orienting reaction). Orienting reaction and voluntary heading to natural acoustic stimulation were observed with relatively short stimuli (500 ms) in Goossens and Van Opstal (1999). In contrast, in our experiment with HRTF spatial rendering, heading toward the target was little observed with short sound stimuli. However, Goosens and Van Opstal authors suggested that head movements could provide spatial information about rich and long enough sounds that would be used by the auditory system to update the internal representation of the target. Our results suggest indeed that the accuracy of pointing to long stimuli could be due to the contribution of heading toward the target providing a more accurate frame of reference for the anticipated control of pointing. However, this does not exclude a direct role of the on-going presentation of the acoustic target.

### Target directions and characteristics of movements

The estimated direction of targets are characterized by a perceived space wider than the real one. This was also observed with hand pointing toward “natural” sounds produced by loudspeakers (Pinek and Brouchon, 1992). However, it was much larger in our study than that observed with natural sounds (less than 10° for Pinek and Brouchon) and this could originate in the use of non-individual HRTF in which interaural differences are not adapted to the geometry of the head. The observed left bias in direction for straight ahead targets could result from a pseudo-neglect effect favoring the left hemispace similar to the pseudo-neglect effect observed with vision (Sosa et al., 2010).

The left/right asymmetry observed in the trajectories kinematics can be explained by this effect as well. Indeed, average and peak velocities increased for targets on the right without effect of the conditions. Along with longer distances covered and fewer number of acceleration peaks, this effect might have caused variations in the control parameters of the movements between the two hemispaces. The left bias observed for the 0° target sound supports this hypothesis. Nevertheless, considering the starting position of the task with the palm put at the center of the set-up, these results could also be accounted for subject's ease to point on the right with their right hand.

### Modularity

This study addresses also the question of the cooperation between different modular neural processes involved in the multisensory and motor representations of targets in goal-directed movements. Do these different processes share a global amodal spatial representation (e.g., Pouget et al., 2002) or do they have their own dedicated spatial representation? Visual and auditory modules use certainly very different reference frames. Sounds are localized thanks to spectral and binaural cues naturally linked to a head-centered frame of reference when visual positions are primarily coded in an eye-centered reference frame. In addition, the visual system is retinotopic whereas the auditory system is characterized by broad tuning and lack of topographical organization (Maier and Groh, 2009).

The question of modularity in motor control arises when we consider the coordination between head orienting movements and hand movements. In the longer sound condition, the auditory stimulation is long enough to allow the triggering of head rotations. Since the amount of rotation of the head is related to the response of participants, there should certainly be a way for the two processes to share common information. This suggests that the heading direction is coded in a body-centered reference frame and can be used directly by the reaching motor command that shares the same reference frame.

To conclude, it is known that sound localization requires the integration of multisensory information and processing of self-generated movements: a stable representation of an auditory source has to be based on acoustic inputs and their relation to motor states (Aytekin et al., 2008). Our results highlight that auditory representations extracted from a sound signal can be transformed online into a sequence of motor commands for coordinated action, underlying the role of the auditory-motor loop in spatial processing.

### Conflict of interest statement

The authors declare that the research was conducted in the absence of any commercial or financial relationships that could be construed as a potential conflict of interest.
